# Climate Anxiety, Loneliness and Perceived Social Isolation

**DOI:** 10.3390/ijerph192214991

**Published:** 2022-11-14

**Authors:** André Hajek, Hans-Helmut König

**Affiliations:** Department of Health Economics and Health Services Research, University Medical Center Hamburg-Eppendorf, Hamburg Center for Health Economics, 20246 Hamburg, Germany

**Keywords:** climate anxiety, climate change, loneliness, social isolation, social exclusion, eco-anxiety, anxiety, fear, concerns, social disconnectedness

## Abstract

Aim: The goal of this study was to investigate the association of climate anxiety with loneliness and perceived social isolation (also stratified by age group). Methods: Data were taken from the general adult population aged 18 to 74 years (n = 3091). Data collection took place in March 2022. Climate anxiety was measured using the Climate Anxiety Scale. The De Jong Gierveld tool was used to quantify loneliness and the Bude and Lantermann tool was used to assess perceived social isolation. Results: Multiple linear regressions revealed an association between higher climate anxiety and higher loneliness (β = 0.06, *p* < 0.001) as well as higher perceived social isolation (β = 0.10, *p* < 0.001) among the total sample. A similar picture was identified in age-stratified regressions (i.e., among individuals aged 18 to 29 years, 30 to 49 years, and among individuals aged 50 to 64 years). However, climate anxiety was neither associated with loneliness nor with perceived social isolation among individuals aged 65 to 74 years. Conclusions: Our current study adds first evidence regarding the link between climate anxiety and loneliness as well as perceived social isolation and can serve as a basis for upcoming studies.

## 1. Introduction

Increased air pollution, extreme temperatures, melting of the polar ice caps, severe storms, floods, seasonal haze, and rising sea levels indicate that a climate change is taking place. The ongoing climate change is a very important concern for the well-being of individuals and the survival of humanity [1]. For these reasons, it appears very plausible that human beings can develop climate anxiety.

The fears referring to challenges in ecology have been mainly discussed in the past 15 years [2] (for a discussion of the early works, please see Cossman [3]). Different kinds of anxiety related to climate change have been described and discussed [4]. For example, the media often referred to eco-anxiety or climate anxiety [4]. Climate anxiety is the type of eco-anxiety which is presumably most heavily discussed [2], especially in the context of teenagers or young adults participating in actions related to climate. Eco-anxiety is often defined rather broadly as a “generalized sense that the ecological foundations of existence are in the process of collapse” (p. 250) [5]. In contrast, climate anxiety can be defined as “anxiety which is significantly related to anthropogenic climate change” (p. 3) [2]. An in-depth review of the terminology has recently been provided [6].

Thus far, a few studies have examined the determinants of climate anxiety and, among other things, demonstrated a negative association between age and climate anxiety [7]. A second example: It has been shown that climate anxiety is higher among female individuals based on a quota-sample (n = 1011 individuals aged 18 to 69 years) from Germany performed in July 2020 [8]. A recent systematic emphasized, however, that various previous studies dealing with climate anxiety relied on rather specific samples focusing on comparably young individuals (such as student samples or based on Amazon MTurk) [9] which questions the generalizability of these findings particularly related to the general adult population. Furthermore, the majority of studies were conducted in the United States [9]. Moreover, it remains completely unclear whether climate anxiety is important for loneliness and perceived social isolation. More precisely, studies examining these associations are completely missing.

Loneliness refers to the difference between actual and desired social relations [10] and perceived social isolation refers to the feeling that one is excluded from society [11]. They are both important for chronic conditions (such as certain physical illnesses) [12] and longevity [13].

Thus, it is important to clarify the link between climate anxiety and loneliness as well as perceived social isolation. Hence, as the very first study, the aim of our study was to investigate the association between climate anxiety and loneliness as well as perceived social isolation (also stratified by age group) in the general German adult population. Our two key hypotheses are as follows: (1) Higher climate anxiety is associated with higher levels of loneliness. (2) Higher climate anxiety is associated with higher levels of perceived social isolation. 

Overall, we think that this knowledge is relevant for clinicians, policymakers and public health staff. For example, it is important for public health staff and policymakers to address individuals at risk for high loneliness or isolation levels (e.g., by using campaigns). Moreover, such knowledge is important for clinicians because as mentioned above loneliness and perceived social isolation are associated with subsequent morbidity and mortality.

The Terror Management Theory by Greenberg and colleagues [14] can act as theoretical background for the association of interest. Uncertainty and concerns regarding natural disasters can be signs of high climate anxiety. Following this theory, the discomfort caused by such uncertainty can cause feelings of stress and worry (to mitigate death anxiety) [15]. These factors could potentially contribute to feelings of loneliness and social isolation. Another possible link between climate anxiety and loneliness/social isolation may be that a high climate anxiety reflects caring for climate change. Thus, individuals who have climate anxiety scores may feel cut off from the rest of society which, in their opinion, may not care as much about the effects of climate change as they do. Additionally, it may be the case that individuals with high climate anxiety levels have already experienced the effects of climate change on a personal level (e.g., via floods). They could feel isolated from individuals living in other parts which were not directly affected by natural disasters. A further explanation is that poor mental health, which can lead to increased levels of loneliness and perceived social isolation, might be attributed to higher levels of climate anxiety [15].

## 2. Materials and Methods

### 2.1. Sample

We used cross-sectional information obtained from a quota-based online survey of 3091 individuals residing in Germany. The age of the respondents ranged from 18 to 74 years. Data were collected between 15 and 21 March 2022. On the selection of the sample: The renowned market research company Bilendi and respondi (Paris, France), an ISO 26362 certified online sampling service, invited the participants. They were selected from a quota-based online sample to ensure that the age, gender, and federal state distribution of the respondents were representatives of the adult German population as a whole. A total of 11,900 people were invited to participate (i.e., the response rate was about 26%). The reasons for non-participation were not listed. Since this was an online sample, a selectivity analysis could not be calculated.

The topics and objectives of the survey were explained in detail prior to the start of the survey. Moreover, before the start of the survey, each individual gave their informed consent (via agreeing to the online consent form) a procedure that is very common in online surveys. The Psychological Ethics Committee of the University Medical Center Hamburg-Eppendorf (LPEK-0412) approved this study.

### 2.2. Dependent Variables

Loneliness was measured using the De Jong Gierveld loneliness [16] scale consisting of six items. Three items were recorded because they were worded positively (unlike the other three remaining items). By averaging the items, a score was developed (ranging from 1 to 4, with higher values reflecting higher loneliness levels). This tool has favorable psychometric properties [16,17] and is widely used. For example, good construct validity has been shown in former research [18,19,20,21]. In our study, Cronbach’s alpha was 0.83 (McDonald’s omega: 0.82).

Perceived social isolation was assessed using a tool created by Bude and Lantermann [11]. It consists of four items. Again, by averaging the items, a final score was computed (1 to 4, higher values indicate higher perceived social isolation). Cronbach’s alpha was 0.91 in our study (McDonald’s omega: 0.91).

### 2.3. Key Independent Variable

To assess climate anxiety, the Climate Anxiety Scale was used. It was developed by Clayton and Karazsia [22]. It comprises 13 items with response options ranging from 1 (strongly disagree) to 7 (strongly agree). The validated [8] German version was used. An overall climate anxiety score was generated using the items’ mean (from 1 to 7, with higher scores indicating higher levels of climate anxiety). In our study, Cronbach’s alpha equaled 0.95 (McDonald’s omega: 0.95).

### 2.4. Covariates

Based on former research in the area of climate anxiety (e.g., [23]), covariates were included in our regression model.

Regarding sociodemographic covariates in regression analysis, we included: sex (men; women; diverse), age, family situation (married, cohabiting with spouse; married, not cohabiting with spouse; divorced; widowed; single), having children in own household (no; yes), state (16 federal states from Germany), migration background (no; yes), level of school education (upper secondary school; qualification for applied upper secondary school; polytechnic Secondary School; intermediate Secondary School; currently in school training/education; without school-leaving qualification/Lower Secondary School), employment situation (full-time employed; retired; other).

Regarding lifestyle-related covariates, we included in regression analysis: smoking status (yes, daily; yes, sometimes; no, not anymore; never smoker), sports activities (no sports activity; less than one hour a week; regularly, 1–2 h a week; regularly, 2–4 h a week; regularly, more than 4 h a week), alcohol intake (daily; several times per week; once a week; 1–3 times per month; less often; never) and having pets (yes, I have pets; no, I do not have pets).

Regarding health-related covariates, in regression, we included: self-rated health (from very poor (1) to very good (5)) and chronic illnesses (no chronic illness; one or more chronic illnesses). Regarding psychological covariates, coronavirus anxiety was included in regression analysis as a covariate. The Coronavirus Anxiety Scale [24] (translated version [25]) was used to this end. It has five items. The sum score varies from 0 to 20 (higher scores reflect more coronavirus anxiety). In our study, Cronbach’s alpha was 0.92 (McDonald’s omega: 0.92).

### 2.5. Statistical Analysis

First, sample characteristics are calculated (also stratified by age group). They were also displayed stratified by age group to attain a better understanding of the specific age groups. This is important because in accordance with our aim outlined in the introduction section regressions were additionally stratified by age group.

Effect sizes (in terms of Pearson’s r) are calculated for the key associations. Commonly, (in absolute terms) Pearson’s r can be interpreted as follows [26]: from 0.10 to 0.29: “small correlation”, from 0.30 to 0.49: “medium correlation”, 0.50 to 1.0: “large correlation”. Thereafter, multiple linear regressions were calculated (1: with loneliness as the outcome; 2: with perceived social isolation as the outcome). Climate anxiety served as the key independent variable. It was adjusted for several factors mentioned in the section “Covariates”. We also calculated effect sizes for the regressions (in terms of eta-squared and partial-eta squared). Such values can be interpreted as follows [26]: 0.01 as “small”, 0.06 as “medium, and 0.14 as “large”.

In a rather explorative manner, age-stratified regressions were conducted in this study. We speculatively assume that the link between climate anxiety and loneliness as well as perceived social isolation is particularly pronounced among younger adults because reflecting on climate may be an important part of life in this age bracket (which can also contribute to social relations). In contrast, we assume that these associations are less pronounced among older adults where other life domains may be of greater importance.

McDonald’s omega was computed using “omegacoef” developed by Shaw [27]. The significance level was set at *p* < 0.05. Stata 16.1 (Stata Corp., College Station, TX, USA) was used for statistical analyses.

## 3. Results

### 3.1. Sample Characteristics

In our total sample, the mean age was 46.5 years (18 to 74 years, SD: 15.3 years). About 50.3% of the individuals were male. The mean climate anxiety score was 2.0 (SD: 1.2). Moreover, the mean loneliness score was 2.1 (SD: 0.7), and mean perceived social isolation score was 1.9 (SD: 0.8). Additional details are given in Table 1.

The association between climate anxiety and loneliness was r = 0.19 (*p* < 0.001). Furthermore, the association between climate anxiety and perceived social isolation was r = 0.28 (*p* < 0.001). Moreover, it may be worth noting that the association between loneliness and perceived social isolation was r = 0.61 (*p* < 0.001).

Among individuals aged 18 to 29 years, the association between climate anxiety and loneliness was r = 0.23, *p* < 0.001 (climate anxiety and social isolation: r = 0.32, *p* < 0.001). Among individuals aged 30 to 49 years, the association between climate anxiety and loneliness was r = 0.21, *p* < 0.001 (climate anxiety and social isolation: r = 0.29, *p* < 0.001). Among individuals aged 50 to 64 years, the association between climate anxiety and loneliness was r = 0.16, *p* < 0.001 (climate anxiety and social isolation: r = 0.22, *p* < 0.001). Lastly, among individuals aged 65 to 74 years, the association between climate anxiety and loneliness was r = 0.11, *p* = 0.02 (climate anxiety and social isolation: r = 0.16, *p* < 0.001).

### 3.2. Regression Analysis

Findings of multiple linear regressions are provided in Table 2 (total sample and stratified by age group). The R^2^ values ranged from 0.21 to 0.26 (with loneliness as the outcome measure) and varied from 0.23 to 0.29 (with perceived social isolation as the outcome measure). Unstandardized beta coefficients are given in this section.

After adjusting for numerous covariates, regressions revealed an association between higher climate anxiety and higher loneliness scores among the total sample (β = 0.06, *p* < 0.001). Very similar findings (in terms of effect size and significance) were obtained among individuals aged 18 to 29 years, individuals aged 30 to 49 years, and among individuals aged 50 to 64 years. However, climate anxiety was not significantly associated with loneliness among individuals aged 65 to 74 years.

Regressions also revealed an association between higher climate anxiety and higher perceived social isolation among the total sample (β = 0.10, *p* < 0.001). Comparable findings were obtained among individuals aged 18 to 29 years, individuals aged 30 to 49 years and among individuals aged 50 to 64 years. In contrast, there was no significant association between climate anxiety and perceived social isolation among individuals aged 65 to 74 years.

In Supplementary Materials, the effect sizes (eta-squared and partial eta-squared values) for all ten multiple linear regressions are shown. For example, in our regression model (with perceived social isolation as an outcome measure and among the total sample), the overall eta-squared value was 24.9% (95% CI: 21.3–26.3%). In this model, the partial eta-squared value for climate anxiety was 2.2% (95% CI: 1.3–3.4%). Further details are given in Supplementary Materials.

Moreover, in Supplementary Materials, multiple linear regressions are shown where we displayed unstandardized and standardized beta-coefficients for all covariates. Please see Supplementary Materials for these details.

## 4. Discussion

The goal of this study was to investigate the association between climate anxiety and loneliness as well as perceived social isolation (also stratified by age group) using data from the general German adult population. Pairwise correlations showed the following associations: climate anxiety and loneliness were positively associated (small effect size) and climate anxiety and perceived social isolation were also positively associated (nearly medium effect size). However, the pairwise correlations should not be overinterpreted since they reflect unadjusted associations. More importantly, regressions showed an association between higher climate anxiety and higher loneliness as well as higher perceived social isolation among the total sample. Comparable findings were identified in age-stratified regressions (i.e., among individuals aged 18 to 29 years, 30 to 49 years and among individuals aged 50 to 64 years). In contrast, climate anxiety was neither associated with loneliness nor with perceived social isolation among individuals aged 65 to 74 years. The effect sizes in terms of partial eta-squared for climate anxiety in all regressions were small.

Our findings are difficult to compare with previous studies since this is the very first study examining the association between these factors. Therefore, our study adds first insights into such associations and can serve as a basis for future research. Prior research showed the importance of age for climate anxiety [28]; this is in accordance with our study.

As outlined in the introduction section, our findings can be explained by using the Terror Management Theory. According to it, emotions of uneasiness evoked by insecurity due to climate change can result in distress and concerns [15] factors which are associated with loneliness and perceived social isolation [29,30]. Another way to explain the association between climate anxiety and higher loneliness and perceived social isolation is as follows: Individuals who score high in climate anxiety may particularly care for climate change. They may feel excluded from other parts of the societies who in their views do not care so much about the consequences of climate change. Moreover, individuals who score high in climate anxiety may already have been directly affected by the impacts of climate change (e.g., in Germany: directly affected individuals or survivors of the severe floods which took place in some parts of Europe including certain parts of Germany in July 2021). They may feel left alone or excluded from individuals living in other regions of Germany (which were not directly affected by natural disasters). Another way to explain the associations of interest is that higher climate anxiety can contribute to poor mental health [15] which in turn can contribute to higher loneliness and higher perceived social isolation.

While our regressions showed quite comparable findings among the different age groups, there was no significant association between climate anxiety and the outcomes among individuals aged 65 to 74 years. Individuals in this age bracket may also fear the consequences of climate change. However, we particularly assume that individuals in this age bracket have other areas of life that deserve a much higher priority (such as family life, caring for grandchildren, caring for established and solid social relationships, or hobbies in retirement that were neglected in working life). Thus, climate anxiety may not reflect a burden (in terms of loneliness or perceived social isolation) for individuals aged 65 to 74 years. Another way to explain such findings may be that the fear of passing away outweighs concerns about climate change among older adults. Moreover, the belief among older adults that one will pass away soon and thus is not significantly impacted by climate change can assist in explaining our findings. However, future research in this area is urgently required (e.g., also based on qualitative approaches e.g., to better understand the underlying reasons for (non-)associations).

Some strengths and limitations of this study are worth noting. A large quota-based sample of the general adult population of Germany (in terms of sex, state, and age group) was employed. Several covariates were included in our regression model. Climate anxiety, loneliness, and perceived social isolation were all assessed using established instruments. Due to the cross-sectional nature of our study, it is challenging to determine the directionality. Moreover, the question was only available in the German language. Additionally, the existence of an online bias cannot be ruled out. Thus, some groups may be underrepresented (for example, individuals with poor German language skills, individuals without access to the internet, or individuals living in institutionalized settings).

## 5. Conclusions

Our current study adds first evidence regarding the link between climate anxiety and loneliness as well as perceived social isolation and can serve as a basis for upcoming studies. Future research in this area is needed (e.g., in other countries or based on qualitative approaches). Moreover, potential mediating and moderating factors (e.g., sex) should be further explored.

## Figures and Tables

**Table 1:** Sample characteristics for the total sample (n = 3091) and stratified by age group.

	Age Group					
Variables	18 to 29 Years	30 to 49 Years	50 to 64 Years	65 to 74 Years	Total Sample	*p*-Value
N (%)	577 (18.7)	1076 (34.8)	995 (32.2)	443 (14.3)	3091 (100.0)	
Sex: N (%)						<0.001
Male	123 (21.3)	506 (47.0)	594 (59.7)	331 (74.7)	1554 (50.3)	
Female	453 (78.5)	567 (52.7)	399 (40.1)	112 (25.3)	1531 (49.5)	
Diverse	1 (0.2)	3 (0.3)	2 (0.2)	0 (0.0)	6 (0.2)	
Age: Mean (SD)	24.5 (3.3)	39.3 (5.5)	56.9 (4.1)	69.0 (2.8)	46.5 (15.3)	<0.001
Children in own household: N (%)						<0.001
No	439 (76.1)	537 (49.9)	775 (77.9)	407 (91.9)	2158 (69.8)	
Yes	138 (23.9)	539 (50.1)	220 (22.1)	36 (8.1)	933 (30.2)	
Marital status: N (%)						<0.001
Married, not cohabiting with spouse; single; divorced; widowed	308 (53.4)	378 (35.1)	416 (41.8)	164 (37.0)	1266 (41.0)	
Married, cohabiting with spouse	269 (46.6)	698 (64.9)	579 (58.2)	279 (63.0)	1825 (59.0)	
State: N (%)						0.052
Baden-Wuerttemberg	81 (14.0)	152 (14.1)	134 (13.5)	50 (11.3)	417 (13.5)	
Bavaria	95 (16.5)	201 (18.7)	144 (14.5)	57 (12.9)	497 (16.1)	
Berlin	17 (2.9)	54 (5.0)	41 (4.1)	26 (5.9)	138 (4.5)	
Brandenburg	11 (1.9)	30 (2.8)	30 (3.0)	20 (4.5)	91 (2.9)	
Bremen	7 (1.2)	9 (0.8)	7 (0.7)	2 (0.5)	25 (0.8)	
Hamburg	12 (2.1)	27 (2.5)	22 (2.2)	9 (2.0)	70 (2.3)	
Hesse	51 (8.8)	67 (6.2)	73 (7.3)	42 (9.5)	233 (7.5)	
Mecklenburg-Western Pomerania	8 (1.4)	19 (1.8)	20 (2.0)	11 (2.5)	58 (1.9)	
Lower Saxony	49 (8.5)	103 (9.6)	88 (8.8)	58 (13.1)	298 (9.6)	
North Rhine-Westphalia	135 (23.4)	208 (19.3)	236 (23.7)	89 (20.1)	668 (21.6)	
Rhineland-Palatinate	35 (6.1)	50 (4.6)	46 (4.6)	18 (4.1)	149 (4.8)	
Saarland	5 (0.9)	15 (1.4)	13 (1.3)	3 (0.7)	36 (1.2)	
Saxony	25 (4.3)	50 (4.6)	49 (4.9)	22 (5.0)	146 (4.7)	
Saxony-Anhalt	13 (2.3)	25 (2.3)	23 (2.3)	18 (4.1)	79 (2.6)	
Schleswig-Holstein	17 (2.9)	38 (3.5)	40 (4.0)	13 (2.9)	108 (3.5)	
Thuringia	16 (2.8)	28 (2.6)	29 (2.9)	5 (1.1)	78 (2.5)	
Migration background: N (%)						<0.001
No migration background	459 (79.5)	917 (85.2)	929 (93.4)	416 (93.9)	2721 (88.0)	
Migration background	118 (20.5)	159 (14.8)	66 (6.6)	27 (6.1)	370 (12.0)	
Education: N (%)						<0.001
Upper secondary school	334 (57.9)	483 (44.9)	284 (28.5)	133 (30.0)	1234 (39.9)	
Qualification for applied upper secondary school	74 (12.8)	142 (13.2)	94 (9.4)	46 (10.4)	356 (11.5)	
Polytechnic Secondary School	5 (0.9)	31 (2.9)	114 (11.5)	46 (10.4)	196 (6.3)	
Intermediate Secondary School	124 (21.5)	345 (32.1)	360 (36.2)	127 (28.7)	956 (30.9)	
Lower Secondary School/Without school-leaving qualification	27 (4.7)	74 (6.9)	142 (14.3)	90 (20.3)	333 (10.8)	
Currently in school training/education	13 (2.3)	1 (0.1)	1 (0.1)	1 (0.2)	16 (0.5)	
Employment status: N (%)						<0.001
Full-time employed	209 (36.2)	645 (59.9)	488 (49.0)	23 (5.2)	1365 (44.2)	
Retired	1 (0.2)	39 (3.6)	215 (21.6)	391 (88.3)	646 (20.9)	
Other	367 (63.6)	392 (36.4)	292 (29.3)	29 (6.5)	1080 (34.9)	
Pets: N (%)						<0.001
Yes, I have pets	252 (43.7)	529 (49.2)	464 (46.6)	147 (33.2)	1392 (45.0)	
No, I do not have pets	325 (56.3)	547 (50.8)	531 (53.4)	296 (66.8)	1699 (55.0)	
Smoking status: N (%)						<0.001
Yes, daily	81 (14.0)	261 (24.3)	298 (29.9)	82 (18.5)	722 (23.4)	
Yes, sometimes	60 (10.4)	89 (8.3)	66 (6.6)	23 (5.2)	238 (7.7)	
No, not anymore	117 (20.3)	285 (26.5)	325 (32.7)	216 (48.8)	943 (30.5)	
Never smoker	319 (55.3)	441 (41.0)	306 (30.8)	122 (27.5)	1188 (38.4)	
Alcohol consumption: N (%)						<0.001
Daily	15 (2.6)	39 (3.6)	81 (8.1)	64 (14.4)	199 (6.4)	
Several times a week	58 (10.1)	173 (16.1)	215 (21.6)	98 (22.1)	544 (17.6)	
Once a week	94 (16.3)	185 (17.2)	130 (13.1)	57 (12.9)	466 (15.1)	
1–3 times a month	139 (24.1)	194 (18.0)	151 (15.2)	61 (13.8)	545 (17.6)	
Less often	149 (25.8)	287 (26.7)	227 (22.8)	83 (18.7)	746 (24.1)	
Never	122 (21.1)	198 (18.4)	191 (19.2)	80 (18.1)	591 (19.1)	
Sports activities: N (%)						<0.001
No sports activity	94 (16.3)	226 (21.0)	352 (35.4)	166 (37.5)	838 (27.1)	
Less than one hour a week	114 (19.8)	213 (19.8)	166 (16.7)	82 (18.5)	575 (18.6)	
Regularly, 1–2 h a week	157 (27.2)	306 (28.4)	221 (22.2)	87 (19.6)	771 (24.9)	
Regularly, 2–4 h a week	116 (20.1)	184 (17.1)	126 (12.7)	64 (14.4)	490 (15.9)	
Regularly, more than 4 h a week	96 (16.6)	147 (13.7)	130 (13.1)	44 (9.9)	417 (13.5)	
Chronic diseases: N (%)						<0.001
Absence of at least one chronic disease	415 (71.9)	705 (65.5)	404 (40.6)	149 (33.6)	1673 (54.1)	
Presence of at least one chronic disease	162 (28.1)	371 (34.5)	591 (59.4)	294 (66.4)	1418 (45.9)	
Self-rated health (from 1 = very bad to 5 = very good): Mean (SD)	3.9 (0.8)	3.8 (0.8)	3.4 (0.9)	3.3 (0.9)	3.6 (0.9)	<0.001
Coronavirus anxiety scale (from 0 to 20, with higher values reflecting higher coronavirus anxiety): Mean (SD)	2.1 (3.7)	1.7 (3.4)	1.0 (2.4)	0.9 (2.3)	1.4 (3.1)	<0.001
Climate anxiety score (from 1 to 7, with higher values reflecting higher climate anxiety): Mean (SD)	2.4 (1.3)	2.1 (1.3)	1.8 (1.0)	1.8 (1.0)	2.0 (1.2)	<0.001
Loneliness (from 1 to 4, with higher values reflecting higher loneliness scores): Mean (SD)	2.1 (0.6)	2.1 (0.6)	2.1 (0.7)	2.0 (0.6)	2.1 (0.7)	0.018
Perceived social isolation (from 1 to 4, with higher values reflecting higher perceived social isolation scores): Mean (SD)	2.2 (0.8)	2.0 (0.8)	1.8 (0.8)	1.8 (0.7)	1.9 (0.8)	<0.001

Notes: Oneway ANOVAs or Chi^2^-tests were conducted, as appropriate (*p*-values).

**Table 2:** Climate anxiety and loneliness as well as perceived social isolation (total sample and stratified by age group). Results of multiple linear regressions.

Independent Variables	Loneliness					Perceived Social Isolation				
	Total Sample	18 to 29 Years	30 to 49 Years	50 to 64 Years	65 to 74 Years	Total Sample	18 to 29 Years	30 to 49 Years	50 to 64 Years	65 to 74 Years
Climate anxiety	0.06 ***	0.06 **	0.06 ***	0.08 ***	0.01	0.10 ***	0.12 ***	0.09 ***	0.11 ***	0.05
	(0.01)	(0.02)	(0.02)	(0.02)	(0.03)	(0.01)	(0.03)	(0.02)	(0.03)	(0.04)
Covariates	✓	✓	✓	✓	✓	✓	✓	✓	✓	✓
R^2^	0.21	0.24	0.24	0.23	0.26	0.25	0.24	0.29	0.25	0.23
Observations	3091	577	1076	995	443	3091	577	1076	995	443

Unstandardized beta-coefficients are reported; robust standard errors in parentheses; *** *p* < 0.001, ** *p* < 0.01; Covariates include (✓) sex, age, family status, presence of children in own household, state, migration background, school education, labor force participation, pet ownership, smoking status, alcohol consumption, frequency of sports activities, self-rated health, chronic conditions, and coronavirus anxiety.

## Data Availability

The datasets generated and/or analysed during the current study are not publicly available due to legal restrictions but are available from the corresponding author on reasonable request.

## Supplementary Materials

The following supporting information can be downloaded at: https://www.mdpi.com/article/10.3390/ijerph192214991/s1; Table S1: Effect sizes for linear regression (η2 and partial η2). Loneliness as outcome and among the total sample. Table S2: Effect sizes for linear regression (η2 and partial η2). Loneliness as outcome and among individuals aged 18 to 29 years. Table S3: Effect sizes for linear regression (η2 and partial η2). Loneliness as outcome and among individuals aged 30 to 49 years. Table S4: Effect sizes for linear regression (η2 and partial η2). Loneliness as outcome and among individuals aged 50 to 64 years. Table S5: Effect sizes for linear regression (η2 and partial η2). Loneliness as outcome and among individuals aged 65 to 74 years. Table S6: Effect sizes for linear regression (η2 and partial η2). Perceived social isolation as outcome and among the total sample. Table S7: Effect sizes for linear regression (η2 and partial η2). Perceived social isolation as outcome and among individuals aged 18 to 29 years. Table S8: Effect sizes for linear regression (η2 and partial η2). Perceived social isolation as outcome and among individuals aged 30 to 49 years. Table S9: Effect sizes for linear regression (η2 and partial η2). Perceived social isolation as outcome and among individuals aged 50 to 64 years. Table S10: Effect sizes for linear regression (η2 and partial η2). Perceived social isolation as outcome and among individuals aged 65 to 74 years. Table S11: Climate anxiety and loneliness as well as perceived social isolation (total sample and stratified by age group). Results of multiple linear regressions—additionally displaying all covariates; Table S12: Climate anxiety and loneliness as well as perceived social isolation (total sample and stratified by age group). Results of multiple linear regressions—additionally displaying all covariates and reporting standardized beta-coefficients
