# Supplementary material for: Climate Anxiety, Loneliness and Perceived Social Isolation
Source: Int J Environ Res Public Health. 2022 Nov 14;19(22):14991. doi: 10.3390/ijerph192214991 (PMC9690346; doi:10.3390/ijerph192214991)
Supplement: Supplementary file 1 [file ijerph-19-14991-s001.zip › ijerph-1990955-supplementary.pdf]

Supplementary Table S1. Effect sizes for linear regression (η2 and partial η2). Loneliness as outcome and among the total sample.

Effect sizes for linear models

Source	Eta-Squared	df	[95% Conf.	Interval]
Model	.2056581	45	.1700038	.2184822
Climate anxiety	.0111321	1	.0049388	.0196738
Sex	.0015356	2	.	.0050993
Age	.0009082	1	.	.0042917
Children in own household	.0010962	1	.	.004688
Marital status	.0475893	1	.0339604	.0630414
State	.0082465	15	.	.0104377
Migration	.0010667	1	.	.0046271
Highest education	.000869	5	.	.0021763
Employment status	.0008849	2	.	.0037509
Smoking	.0008049	3	.	.0030583
Alcohol intake	.0014469	5	.	.0035252
Sports activities	.0071167	4	.001657	.0130855
Chronic conditions	.000793	1	.	.004038
Self-rated health	.0571137	1	.0422279	.0736932
Coronavirus anxiety	.0183523	1	.0100986	.0288468
Pet ownership	.0005758	1	.	.0035282

Note: Eta-Squared values for individual model terms are partial.

Supplementary Table S2. Effect sizes for linear regression (η2 and partial η2). Loneliness as outcome and among individuals aged 18 to 29 years.

Effect sizes for linear models

Source	Eta-Squared	df	[95% Conf.	Interval]
Model	.2397015	45	.1171502	.237014
Climate anxiety	.014102	1	.0011521	.0401476
Sex	.0008805	2	.	.0088315
Age	.0020926	1	.	.0167724
Children in own household	.0071142	1	.	.0279265
Marital status	.032297	1	.0092566	.0669461
State	.02829	15	.	.0309132
Migration	.0058139	1	.	.0253616
Highest education	.0027976	5	.	.0057003
Employment status	.0019239	2	.	.0129972
Smoking	.008064	3	.	.0246203
Alcohol intake	.0054243	5	.	.0134377
Sports activities	.0120314	4	.	.0294877
Chronic conditions	.0000122	1	.	.0035293
Self-rated health	.0710329	1	.0346213	.1160761
Coronavirus anxiety	.0115089	1	.0004565	.0358365
Pet ownership	.0015626	1	.	.0152246

Note: Eta-Squared values for individual model terms are partial.

Supplementary Table S3. Effect sizes for linear regression ($\eta 2$ and partial $\eta 2$). Loneliness as outcome and among individuals aged 30 to 49 years.

Effect sizes for linear models

Source	Eta-Squared	df	[95% Conf.	Interval]
Model	.244021	45	.1685354	.2546706
Climate anxiety	.0126666	1	.0026887	.0294962
Sex	.0005071	2	.	.0048548
Age	.0000632	1	.	.0041142
Children in own household	.0008862	1	.	.0081669
Marital status	.0563092	1	.0320838	.085564
State	.0101275	15	.	.0084188
Migration	.0007124	1	.	.0076198
Highest education	.0017346	5	.	.0039734
Employment status	.0006604	2	.	.005543
Smoking	.0021921	3	.	.0085158
Alcohol intake	.0028776	5	.	.0071851
Sports activities	.0133117	4	.0010258	.0269778
Chronic conditions	.00678	1	.0004479	.0202144
Self-rated health	.0561491	1	.0319594	.0853745
Coronavirus anxiety	.0230956	1	.0084295	.0441602
Pet ownership	.0008531	1	.	.0080661

Note: Eta-Squared values for individual model terms are partial.

Supplementary Table S4. Effect sizes for linear regression ($\eta 2$ and partial $\eta 2$). Loneliness as outcome and among individuals aged 50 to 64 years.

Effect sizes for linear models

Source	Eta-Squared	df	[95% Conf.	Interval]
Model	.2266104	45	.1466088	.2351752
Climate anxiety	.0173301	1	.0047097	.0371625
Sex	.0029254	2	.	.01212
Age	.0013998	1	.	.0100909
Children in own household	.0000715	1	.	.0045101
Marital status	.0497704	1	.0262471	.0790339
State	.0124017	15	.	.0116506
Migration	.0042105	1	.	.0162598
Highest education	.0050542	5	.	.0120653
Employment status	.0027793	2	.	.0117943
Smoking	.00235	3	.	.0091611
Alcohol intake	.0034005	5	.	.0084645
Sports activities	.0076858	4	.	.0183915
Chronic conditions	.0007385	1	.	.0081485
Self-rated health	.0626269	1	.0361023	.09434
Coronavirus anxiety	.0106207	1	.001577	.0271952
Pet ownership	.0001823	1	.	.0056576

Note: Eta-Squared values for individual model terms are partial.

Supplementary Table S5. Effect sizes for linear regression (η2 and partial η2). Loneliness as outcome and among individuals aged 65 to 74 years.

Effect sizes for linear models

Source	Eta-Squared	df	[95% Conf.	Interval]
Model	.2606007	44	.1103761	.2513943
Climate anxiety	.0001307	1	.	.0099512
Sex	.0115726	1	.	.0407655
Age	.01119	1	.	.0400745
Children in own household	.0022814	1	.	.0207803
Marital status	.0520653	1	.0177162	.0998011
State	.0369206	15	.	.039848
Migration	.0084714	1	.	.0349664
Highest education	.0047022	5	.	.010873
Employment status	.0340914	2	.0060429	.0728698
Smoking	.0083583	3	.	.0277443
Alcohol intake	.015754	5	.	.0352399
Sports activities	.0213859	4	.	.0479403
Chronic conditions	.0017151	1	.	.0190159
Self-rated health	.0215964	1	.0024475	.0573446
Coronavirus anxiety	.028764	1	.0052769	.0680763
Pet ownership	.0013774	1	.	.0178473

Note: Eta-Squared values for individual model terms are partial.

Supplementary Table S6. Effect sizes for linear regression (η2 and partial η2). Perceived social isolation as outcome and among the total sample.

Effect sizes for linear models

Source	Eta-Squared	df	[95% Conf.	Interval]
Model	.248738	45	.2127736	.2627555
Climate anxiety	.0225343	1	.0132913	.0339564
Sex	.0000569	2	.	.0008211
Age	.020486	1	.0117132	.0314681
Children in own household	.0003507	1	.	.0029315
Marital status	.01007	1	.0042357	.0182689
State	.0039114	15	.	.0037069
Migration	.0001508	1	.	.0022645
Highest education	.0015217	5	.	.0036819
Employment status	.0032324	2	.0002548	.0080612
Smoking	.0026216	3	.	.0066227
Alcohol intake	.0019081	5	.	.0044535
Sports activities	.0065788	4	.0013591	.0123191
Chronic conditions	.0000213	1	.	.0013957
Self-rated health	.0562467	1	.0414688	.0727298
Coronavirus anxiety	.0418511	1	.0290651	.05654
Pet ownership	.0001749	1	.	.0023592

Note: Eta-Squared values for individual model terms are partial.

Supplementary Table S7. Effect sizes for linear regression (η2 and partial η2). Perceived social isolation as outcome and among individuals aged 18 to 29 years.

Effect sizes for linear models

Source	Eta-Squared	df	[95% Conf.	Interval]
Model	.2400093	45	.1174578	.2373559
Climate anxiety	.034447	1	.0104553	.0698748
Sex	.0052101	2	.	.0214019
Age	.0028285	1	.	.018711
Children in own household	1.73e-06	1	.	.
Marital status	.0010216	1	.	.0134124
State	.01565	15	.	.0088514
Migration	.0001726	1	.	.0086368
Highest education	.0087473	5	.	.0207842
Employment status	.0023358	2	.	.0142752
Smoking	.0043156	3	.	.0165175
Alcohol intake	.0030232	5	.	.0065134
Sports activities	.0092193	4	.	.0242972
Chronic conditions	.0000247	1	.	.0048481
Self-rated health	.0549705	1	.0233127	.0964598
Coronavirus anxiety	.0181015	1	.0025102	.0464637
Pet ownership	.0001683	1	.	.0085837

Note: Eta-Squared values for individual model terms are partial.

Supplementary Table S8. Effect sizes for linear regression (η2 and partial η2). Perceived social isolation as outcome and among individuals aged 30 to 49 years.

Effect sizes for linear models

Source	Eta-Squared	df	[95% Conf.	Interval]
Model	.2923251	45	.2175322	.3052995
Climate anxiety	.0244261	1	.0092548	.0459389
Sex	.0006126	2	.	.0053405
Age	.002282	1	.	.0117017
Children in own household	.0011152	1	.	.0088308
Marital status	.0119485	1	.0023602	.02842
State	.014885	15	.	.0164783
Migration	.0011839	1	.	.0090204
Highest education	.0013962	5	.	.0027755
Employment status	.0055873	2	.	.0168684
Smoking	.0053748	3	.	.0151839
Alcohol intake	.0030396	5	.	.007581
Sports activities	.0158491	4	.0022072	.0307327
Chronic conditions	.0011364	1	.	.0088896
Self-rated health	.0407732	1	.0203883	.066814
Coronavirus anxiety	.077106	1	.0487689	.1096713
Pet ownership	.0000512	1	.	.0038953

Note: Eta-Squared values for individual model terms are partial.

Supplementary Table S9. Effect sizes for linear regression (η2 and partial η2). Perceived social isolation as outcome and among individuals aged 50 to 64 years.

Effect sizes for linear models

Source	Eta-Squared	df	[95% Conf.	Interval]
Model	.2543317	45	.1743393	.2646793
Climate anxiety	.0252496	1	.0092773	.0480639
Sex	.0016414	2	.	.0090065
Age	.0000926	1	.	.0048101
Children in own household	2.30e-06	1	.	.0008386
Marital status	.0224399	1	.0075792	.044273
State	.0139041	15	.	.0142097
Migration	.0007468	1	.	.0081764
Highest education	.0034401	5	.	.0085582
Employment status	.0098472	2	.0006473	.0246702
Smoking	.0020677	3	.	.0084304
Alcohol intake	.0023394	5	.	.0057119
Sports activities	.0100553	4	.	.0223787
Chronic conditions	.0005991	1	.	.0076618
Self-rated health	.0801654	1	.0502024	.1145931
Coronavirus anxiety	.0279577	1	.0109787	.0516537
Pet ownership	.0034367	1	.	.0147269

Note: Eta-Squared values for individual model terms are partial.

Supplementary Table S10. Effect sizes for linear regression (η2 and partial η2). Perceived social isolation as outcome and among individuals aged 65 to 74 years.

Effect sizes for linear models

Source	Eta-Squared	df	[95% Conf.	Interval]
Model	.2341297	44	.0838798	.221281
Climate anxiety	.004813	1	.	.027272
Sex	.0046966	1	.	.0270032
Age	.0041123	1	.	.0256205
Children in own household	.0004172	1	.	.0132177
Marital status	.0049663	1	.	.027623
State	.0362612	15	.	.0388085
Migration	.0211031	1	.0022792	.0565792
Highest education	.0136247	5	.	.031322
Employment status	.0202424	2	.0005433	.0522015
Smoking	.0112803	3	.	.0335795
Alcohol intake	.0293855	5	.	.0574166
Sports activities	.0091618	4	.	.0258811
Chronic conditions	4.89e-07	1	.	.
Self-rated health	.0342285	1	.0078255	.0758727
Coronavirus anxiety	.0102931	1	.	.03843
Pet ownership	.0000427	1	.	.0071014

Note: Eta-Squared values for individual model terms are partial.

Supplementary Table S11. Climate anxiety and loneliness as well as perceived social isolation (total sample and stratified by age group). Results of multiple linear regressions – additionally displaying all covariates

Independent variables	Loneliness					Perceived social isolation				
	Total sample	18 to 29 years	30 to 49 years	50 to 64 years	65 to 74 years	Total sample	18 to 29 years	30 to 49 years	50 to 64 years	65 to 74 years
Climate anxiety	0.06***	0.06**	0.06***	0.08***	0.01	0.10***	0.12***	0.09***	0.11***	0.05
	(0.01)	(0.02)	(0.02)	(0.02)	(0.03)	(0.01)	(0.03)	(0.02)	(0.03)	(0.04)
Sex: - Women (Ref.: Men)	-0.05+	-0.03	0.01	-0.07	-0.15*	-0.01	0.06	0.03	-0.04	-0.12
	(0.02)	(0.07)	(0.04)	(0.04)	(0.07)	(0.03)	(0.08)	(0.05)	(0.05)	(0.09)
- Diverse	0.21***	0.30*	0.24*	0.23+		0.09	1.15***	0.25	-0.47*	
	(0.05)	(0.14)	(0.10)	(0.13)		(0.30)	(0.18)	(0.44)	(0.21)	
Age	-0.00	-0.01	-0.00	-0.01	-0.02*	-0.01***	-0.01	-0.01	-0.00	-0.02
	(0.00)	(0.01)	(0.00)	(0.01)	(0.01)	(0.00)	(0.01)	(0.00)	(0.01)	(0.01)
Children in own household: - Yes (Reference: No)	0.05+	0.14+	0.04	0.01	-0.10	-0.03	-0.00	-0.05	-0.00	-0.05
	(0.03)	(0.07)	(0.04)	(0.05)	(0.08)	(0.03)	(0.09)	(0.05)	(0.05)	(0.12)
Marital status: Married, co-habiting with spouse (Ref.: married, not cohabiting with spouse; divorced; widowed; single)	-0.29***	-0.25***	-0.33***	-0.30***	-0.27***	-0.15***	-0.05	-0.18***	-0.22***	-0.10
	(0.02)	(0.06)	(0.04)	(0.04)	(0.06)	(0.03)	(0.07)	(0.05)	(0.05)	(0.07)
State: - Bavaria (Ref.: Baden-Wuerttemberg)	0.10*	0.08	0.03	0.17*	0.05	0.02	-0.03	-0.01	0.08	-0.03
	(0.04)	(0.09)	(0.06)	(0.08)	(0.12)	(0.05)	(0.11)	(0.08)	(0.09)	(0.14)
- Berlin	0.01	-0.21	-0.04	0.07	-0.02	-0.00	0.03	0.02	-0.11	-0.03
	(0.06)	(0.16)	(0.09)	(0.11)	(0.15)	(0.07)	(0.16)	(0.11)	(0.12)	(0.20)
- Brandenburg	-0.11+	-0.29	-0.01	-0.13	-0.15	-0.02	-0.27	-0.04	0.12	0.06
	(0.06)	(0.18)	(0.10)	(0.11)	(0.17)	(0.08)	(0.19)	(0.11)	(0.16)	(0.21)
- Bremen	0.02	0.27	-0.14	0.00	0.08	-0.09	-0.01	-0.04	-0.30+	0.01
	(0.12)	(0.20)	(0.24)	(0.18)	(0.33)	(0.15)	(0.36)	(0.28)	(0.16)	(0.39)
- Hamburg	0.06	0.02	-0.02	0.08	0.30	0.04	0.00	0.06	-0.00	0.30
	(0.08)	(0.18)	(0.12)	(0.14)	(0.25)	(0.09)	(0.25)	(0.15)	(0.16)	(0.25)
- Hesse	0.09+	0.04	0.07	0.11	0.11	0.04	-0.05	0.07	0.02	0.17
	(0.05)	(0.11)	(0.08)	(0.09)	(0.13)	(0.06)	(0.13)	(0.11)	(0.11)	(0.15)
- Mecklenburg-Western Pomerania	0.03	0.03	-0.09	0.05	0.12	0.11	0.13	-0.07	-0.03	0.47+
	(0.08)	(0.21)	(0.12)	(0.14)	(0.20)	(0.11)	(0.27)	(0.15)	(0.18)	(0.27)
- Lower Saxony	0.04	0.01	0.02	0.07	-0.02	0.03	0.05	-0.05	0.01	0.15
	(0.05)	(0.10)	(0.07)	(0.09)	(0.12)	(0.05)	(0.14)	(0.09)	(0.10)	(0.15)
- North Rhine-Westphalia	0.06	0.07	0.06	0.07	-0.02	0.03	0.09	0.08	-0.04	0.03
	(0.04)	(0.08)	(0.06)	(0.07)	(0.10)	(0.04)	(0.11)	(0.08)	(0.08)	(0.13)
- Rhineland-Palatinate	0.08	0.08	-0.02	0.22*	-0.09	0.05	0.05	-0.09	0.21+	-0.09
	(0.06)	(0.12)	(0.09)	(0.11)	(0.14)	(0.06)	(0.14)	(0.11)	(0.12)	(0.18)
- Saarland	-0.18+	-0.12	-0.25	0.02	-0.32	-0.22+	-0.16	-0.28	-0.24	0.57*
	(0.10)	(0.15)	(0.17)	(0.19)	(0.27)	(0.11)	(0.24)	(0.18)	(0.19)	(0.25)
- Saxony	-0.01	0.16	-0.04	0.06	-0.35*	-0.11+	0.03	-0.17	-0.04	-0.21
	(0.06)	(0.16)	(0.10)	(0.10)	(0.15)	(0.06)	(0.16)	(0.11)	(0.12)	(0.14)
- Saxony-Anhalt	0.04	0.29	-0.05	0.07	-0.12	0.00	0.36+	-0.34**	0.05	0.12
	(0.07)	(0.21)	(0.13)	(0.15)	(0.13)	(0.08)	(0.22)	(0.13)	(0.16)	(0.19)
- Schleswig-Holstein	0.15*	0.31*	0.17	0.11	0.02	0.04	0.04	-0.03	0.16	-0.06
	(0.06)	(0.14)	(0.12)	(0.10)	(0.16)	(0.08)	(0.23)	(0.13)	(0.14)	(0.19)
- Thuringia	0.09	0.10	0.02	0.15	-0.25	-0.01	0.17	-0.06	-0.03	-0.10
	(0.08)	(0.20)	(0.11)	(0.15)	(0.28)	(0.09)	(0.23)	(0.14)	(0.16)	(0.25)
Migration background: - Yes (Ref.: No)	0.06+	0.11+	-0.04	0.16*	0.22+	0.03	0.02	-0.07	0.08	0.42**
	(0.03)	(0.07)	(0.05)	(0.07)	(0.13)	(0.04)	(0.08)	(0.06)	(0.09)	(0.16)
Highest educational degree: - Qualification for Applied Upper Secondary School (Ref.: Upper Secondary School)	0.01	-0.06	0.02	-0.03	0.09	0.06	0.17+	0.01	-0.10	0.21+
	(0.04)	(0.07)	(0.06)	(0.07)	(0.10)	(0.04)	(0.09)	(0.07)	(0.08)	(0.12)
- Polytechnic Secondary School	-0.04	0.14	0.03	-0.06	-0.01	0.08	0.00	0.02	0.07	0.12
	(0.05)	(0.25)	(0.10)	(0.07)	(0.10)	(0.06)	(0.43)	(0.14)	(0.09)	(0.14)
- Intermediate Secondary School	0.03	0.03	0.00	0.01	0.04	0.03	0.03	-0.01	0.03	0.17+
	(0.03)	(0.08)	(0.04)	(0.05)	(0.07)	(0.03)	(0.09)	(0.05)	(0.06)	(0.09)
- Lower Secondary School	0.01	-0.05	0.04	-0.04	0.03	0.07	-0.02	0.09	-0.02	0.20+
	(0.04)	(0.11)	(0.08)	(0.07)	(0.09)	(0.05)	(0.16)	(0.10)	(0.07)	(0.11)
- Currently in school training/education	0.00	-0.01	-0.73***	1.15***	0.59***	0.14	0.27+	-0.21	-0.04	0.01
	(0.14)	(0.15)	(0.21)	(0.13)	(0.18)	(0.10)	(0.14)	(0.24)	(0.16)	(0.20)
Employment status: - Retired (Ref.: Full-time employed)	-0.05	-0.40*	0.06	-0.05	0.40***	0.02	-0.81***	0.27+	-0.04	0.04
	(0.04)	(0.18)	(0.11)	(0.06)	(0.10)	(0.04)	(0.22)	(0.15)	(0.07)	(0.13)
- Other	0.01	-0.05	-0.02	0.05	0.63***	0.10**	0.00	0.07	0.14**	0.45+
	(0.03)	(0.06)	(0.04)	(0.05)	(0.17)	(0.03)	(0.08)	(0.05)	(0.06)	(0.23)
Smoking status: - Yes, daily (Ref.: Never smoker)	-0.04	-0.11	-0.01	-0.06	-0.09	-0.07+	0.00	-0.06	-0.07	-0.17
	(0.03)	(0.08)	(0.05)	(0.05)	(0.09)	(0.04)	(0.10)	(0.06)	(0.06)	(0.11)
- Yes, sometimes	0.01	0.11	0.09	-0.08	-0.15	0.07	0.12	0.14	-0.01	0.10
	(0.04)	(0.10)	(0.07)	(0.08)	(0.13)	(0.05)	(0.12)	(0.08)	(0.09)	(0.20)
- No, not anymore	-0.01	-0.02	0.02	-0.01	-0.12+	-0.03	-0.07	-0.04	0.01	-0.11
	(0.03)	(0.07)	(0.05)	(0.05)	(0.06)	(0.03)	(0.08)	(0.05)	(0.06)	(0.08)
Alcohol consumption: - Daily (Ref.: Never)	0.05	0.02	-0.03	0.12	0.04	0.10	0.18	0.04	0.11	0.11
	(0.05)	(0.22)	(0.12)	(0.08)	(0.10)	(0.07)	(0.28)	(0.16)	(0.10)	(0.13)
- Several times a week	0.00	0.10	-0.08	0.07	-0.09	0.01	-0.02	-0.04	0.06	-0.04
	(0.04)	(0.11)	(0.06)	(0.06)	(0.08)	(0.04)	(0.13)	(0.08)	(0.07)	(0.11)
- Once a week	-0.04	0.01	-0.10	0.01	-0.03	-0.03	0.01	-0.07	0.01	-0.08
	(0.04)	(0.09)	(0.06)	(0.07)	(0.11)	(0.04)	(0.11)	(0.08)	(0.08)	(0.12)
- 1-3 times a month	-0.03	0.02	-0.05	0.03	-0.19+	-0.03	-0.06	-0.04	0.08	-0.32**
	(0.04)	(0.08)	(0.06)	(0.07)	(0.10)	(0.04)	(0.10)	(0.07)	(0.08)	(0.11)
- Less often	0.01	0.10	-0.07	0.08	-0.11	-0.01	0.02	-0.10	0.06	-0.10
	(0.03)	(0.07)	(0.06)	(0.06)	(0.09)	(0.04)	(0.09)	(0.07)	(0.07)	(0.11)

Sports activities: Less than one hour a week (Ref.: No sports activity)	-0.08*	-0.17+	-0.05	-0.06	-0.15+	-0.08*	-0.17	-0.09	-0.08	0.01
	(0.03)	(0.09)	(0.06)	(0.06)	(0.08)	(0.04)	(0.10)	(0.07)	(0.07)	(0.11)
- Regularly, 1-2 hours a week	-0.12***	-0.18*	-0.10+	-0.12*	-0.21**	-0.09*	-0.13	-0.13*	-0.10	-0.09
	(0.03)	(0.09)	(0.05)	(0.06)	(0.08)	(0.04)	(0.10)	(0.07)	(0.06)	(0.09)
- Regularly, 2-4 hours a week	-0.12***	-0.19+	-0.19**	-0.01	-0.13	-0.08*	-0.15	-0.22**	0.11	-0.13
	(0.04)	(0.09)	(0.06)	(0.07)	(0.09)	(0.04)	(0.11)	(0.07)	(0.08)	(0.11)
- Regularly, more than 4 hours a week	-0.13***	-0.12	-0.18**	-0.12+	-0.15	-0.19***	-0.23*	-0.28***	-0.10	-0.19
	(0.04)	(0.10)	(0.07)	(0.07)	(0.11)	(0.04)	(0.12)	(0.08)	(0.07)	(0.12)
Chronic diseases: Presence of at least one chronic disease (Absence of chronic diseases)	-0.04	0.00	-0.11**	0.04	-0.06	-0.01	0.01	-0.06	0.04	-0.00
	(0.02)	(0.06)	(0.04)	(0.04)	(0.07)	(0.03)	(0.08)	(0.05)	(0.05)	(0.08)
Self-rated health (1 = very bad to 5 = very good)	-0.19***	-0.22***	-0.20***	-0.21***	-0.11**	-0.23***	-0.23***	-0.20***	-0.27***	-0.17***
	(0.01)	(0.04)	(0.03)	(0.03)	(0.04)	(0.02)	(0.05)	(0.03)	(0.03)	(0.05)
Coronavirus anxiety (CAS)	0.03***	0.02*	0.03***	0.03***	0.05**	0.05***	0.03**	0.07***	0.05***	0.03*
	(0.00)	(0.01)	(0.01)	(0.01)	(0.01)	(0.00)	(0.01)	(0.01)	(0.01)	(0.02)
Pet ownership: No (Ref.: Yes)	-0.03	-0.05	-0.04	0.02	-0.04	0.02	-0.02	-0.01	0.08+	-0.01
	(0.02)	(0.05)	(0.04)	(0.04)	(0.06)	(0.03)	(0.06)	(0.04)	(0.05)	(0.07)
Constant	2.98***	3.19***	3.13***	3.09***	3.98***	3.01***	3.07***	2.98***	2.59***	3.37***
	(0.09)	(0.30)	(0.20)	(0.33)	(0.73)	(0.11)	(0.34)	(0.25)	(0.36)	(0.90)
Observations	3,091	577	1,076	995	443	3,091	577	1,076	995	443
R^2	0.21	0.24	0.24	0.23	0.26	0.25	0.24	0.29	0.25	0.23

Unstandardized beta-coefficients are reported; robust standard errors in parentheses; *** p<0.001, ** p<0.01, * p<0.05, + p<0.10

Supplementary Table S12. Climate anxiety and loneliness as well as perceived social isolation (total sample and stratified by age group). Results of multiple linear regressions – additionally displaying all covariates and reporting standardized beta-coefficients

Independent variables	Loneliness					Perceived social isolation				
	Total sample	18 to 29 years	30 to 49 years	50 to 64 years	65 to 74 years	Total sample	18 to 29 years	30 to 49 years	50 to 64 years	65 to 74 years
Climate anxiety	0.11	0.13	0.11	0.13	0.01	0.15	0.21	0.15	0.15	0.07
	(0.01)	(0.02)	(0.02)	(0.02)	(0.03)	(0.01)	(0.03)	(0.02)	(0.03)	(0.04)
Sex: - Women (Ref.: Men)	-0.04	-0.02	0.01	-0.05	-0.11	-0.00	0.03	0.02	-0.03	-0.07
	(0.02)	(0.07)	(0.04)	(0.04)	(0.07)	(0.03)	(0.08)	(0.05)	(0.05)	(0.09)
- Diverse	0.01	0.02	0.02	0.02		0.01	0.06	0.02	-0.03	
	(0.05)	(0.14)	(0.10)	(0.13)		(0.30)	(0.18)	(0.44)	(0.21)	
Age	-0.04	-0.05	-0.01	-0.04	-0.10	-0.18	-0.06	-0.04	-0.01	-0.06
	(0.00)	(0.01)	(0.00)	(0.01)	(0.01)	(0.00)	(0.01)	(0.00)	(0.01)	(0.01)
Children in own household: - Yes (Reference: No)	0.03	0.09	0.03	0.01	-0.04	-0.02	-0.00	-0.03	-0.00	-0.02
	(0.03)	(0.07)	(0.04)	(0.05)	(0.08)	(0.03)	(0.09)	(0.05)	(0.05)	(0.12)
Marital status: Married, cohabiting with spouse (Ref.: married, not cohabiting with spouse; divorced; widowed; single)	-0.22	-0.19	-0.25	-0.22	-0.22	-0.09	-0.03	-0.11	-0.14	-0.07
	(0.02)	(0.06)	(0.04)	(0.04)	(0.06)	(0.03)	(0.07)	(0.05)	(0.05)	(0.07)
State: - Bavaria (Ref.: Baden-Wuerttemberg)	0.05	0.05	0.02	0.09	0.03	0.01	-0.02	-0.01	0.04	-0.02
	(0.04)	(0.09)	(0.06)	(0.08)	(0.12)	(0.05)	(0.11)	(0.08)	(0.09)	(0.14)
- Berlin	0.00	-0.06	-0.02	0.02	-0.01	-0.00	0.01	0.00	-0.03	-0.01
	(0.06)	(0.16)	(0.09)	(0.11)	(0.15)	(0.07)	(0.16)	(0.11)	(0.12)	(0.20)
- Brandenburg	-0.03	-0.06	-0.00	-0.03	-0.05	-0.00	-0.05	-0.01	0.03	0.02
	(0.06)	(0.18)	(0.10)	(0.11)	(0.17)	(0.08)	(0.19)	(0.11)	(0.16)	(0.21)
- Bremen	0.00	0.05	-0.02	0.00	0.01	-0.01	-0.00	-0.00	-0.03	0.00
	(0.12)	(0.20)	(0.24)	(0.18)	(0.33)	(0.15)	(0.36)	(0.28)	(0.16)	(0.39)
- Hamburg	0.01	0.01	-0.00	0.02	0.07	0.01	0.00	0.01	-0.00	0.06
	(0.08)	(0.18)	(0.12)	(0.14)	(0.25)	(0.09)	(0.25)	(0.15)	(0.16)	(0.25)
- Hesse	0.03	0.02	0.02	0.04	0.05	0.01	-0.02	0.02	0.01	0.07
	(0.05)	(0.11)	(0.08)	(0.09)	(0.13)	(0.06)	(0.13)	(0.11)	(0.11)	(0.15)
- Mecklenburg-Western Pomerania	0.01	0.01	-0.02	0.01	0.03	0.02	0.02	-0.01	-0.00	0.10
	(0.08)	(0.21)	(0.12)	(0.14)	(0.20)	(0.11)	(0.27)	(0.15)	(0.18)	(0.27)
- Lower Saxony	0.02	0.00	0.01	0.03	-0.01	0.01	0.02	-0.02	0.00	0.07
	(0.05)	(0.10)	(0.07)	(0.09)	(0.12)	(0.05)	(0.14)	(0.09)	(0.10)	(0.15)
- North Rhine-Westphalia	0.04	0.05	0.04	0.05	-0.01	0.02	0.05	0.04	-0.02	0.02
	(0.04)	(0.08)	(0.06)	(0.07)	(0.10)	(0.04)	(0.11)	(0.08)	(0.08)	(0.13)
- Rhineland-Palatinate	0.03	0.03	-0.01	0.07	-0.03	0.01	0.01	-0.02	0.06	-0.02
	(0.06)	(0.12)	(0.09)	(0.11)	(0.14)	(0.06)	(0.14)	(0.11)	(0.12)	(0.18)
- Saarland	-0.03	-0.02	-0.05	0.00	-0.04	-0.03	-0.02	-0.04	-0.04	0.06
	(0.10)	(0.15)	(0.17)	(0.19)	(0.27)	(0.11)	(0.24)	(0.18)	(0.19)	(0.25)
- Saxony	-0.00	0.05	-0.01	0.02	-0.12	-0.03	0.01	-0.04	-0.01	-0.06
	(0.06)	(0.16)	(0.10)	(0.10)	(0.15)	(0.06)	(0.16)	(0.11)	(0.12)	(0.14)
- Saxony-Anhalt	0.01	0.07	-0.01	0.02	-0.04	0.00	0.07	-0.06	0.01	0.03
	(0.07)	(0.21)	(0.13)	(0.15)	(0.13)	(0.08)	(0.22)	(0.13)	(0.16)	(0.19)
- Schleswig-Holstein	0.04	0.08	0.05	0.03	0.01	0.01	0.01	-0.01	0.04	-0.01
	(0.06)	(0.14)	(0.12)	(0.10)	(0.16)	(0.08)	(0.23)	(0.13)	(0.14)	(0.19)
- Thuringia	0.02	0.03	0.01	0.04	-0.04	-0.00	0.04	-0.01	-0.01	-0.01
	(0.08)	(0.20)	(0.11)	(0.15)	(0.28)	(0.09)	(0.23)	(0.14)	(0.16)	(0.25)
Migration background: - Yes (Ref.: No)	0.03	0.07	-0.02	0.06	0.08	0.01	0.01	-0.03	0.02	0.14
	(0.03)	(0.07)	(0.05)	(0.07)	(0.13)	(0.04)	(0.08)	(0.06)	(0.09)	(0.16)
Highest educational degree: - Qualification for Applied Upper Secondary School (Ref.: Upper Secondary School)	0.01	-0.03	0.01	-0.02	0.05	0.02	0.07	0.01	-0.04	0.09
	(0.04)	(0.07)	(0.06)	(0.07)	(0.10)	(0.04)	(0.09)	(0.07)	(0.08)	(0.12)
- Polytechnic Secondary School	-0.01	0.02	0.01	-0.03	-0.01	0.02	0.00	0.00	0.03	0.05
	(0.05)	(0.25)	(0.10)	(0.07)	(0.10)	(0.06)	(0.43)	(0.14)	(0.09)	(0.14)
- Intermediate Secondary School	0.02	0.02	0.00	0.00	0.03	0.02	0.02	-0.01	0.02	0.10
	(0.03)	(0.08)	(0.04)	(0.05)	(0.07)	(0.03)	(0.09)	(0.05)	(0.06)	(0.09)
- Lower Secondary School	0.00	-0.02	0.01	-0.02	0.02	0.03	-0.01	0.03	-0.01	0.11
	(0.04)	(0.11)	(0.08)	(0.07)	(0.09)	(0.05)	(0.16)	(0.10)	(0.07)	(0.11)
- Currently in school training/education	0.00	-0.00	-0.03	0.05	0.05	0.01	0.05	-0.01	-0.00	0.00
	(0.14)	(0.15)	(0.21)	(0.13)	(0.18)	(0.10)	(0.14)	(0.24)	(0.16)	(0.20)
Employment status: - Retired (Ref.: Full-time employed)	-0.03	-0.03	0.02	-0.03	0.21	0.01	-0.04	0.06	-0.02	0.02
	(0.04)	(0.18)	(0.11)	(0.06)	(0.10)	(0.04)	(0.22)	(0.15)	(0.07)	(0.13)
- Other	0.01	-0.04	-0.02	0.03	0.25	0.06	0.00	0.04	0.08	0.15
	(0.03)	(0.06)	(0.04)	(0.05)	(0.17)	(0.03)	(0.08)	(0.05)	(0.06)	(0.23)
Smoking status: - Yes, daily (Ref.: Never smoker)	-0.03	-0.06	-0.01	-0.04	-0.06	-0.04	0.00	-0.03	-0.04	-0.09
	(0.03)	(0.08)	(0.05)	(0.05)	(0.09)	(0.04)	(0.10)	(0.06)	(0.06)	(0.11)
- Yes, sometimes	0.00	0.05	0.04	-0.03	-0.05	0.02	0.05	0.05	-0.00	0.03
	(0.04)	(0.10)	(0.07)	(0.08)	(0.13)	(0.05)	(0.12)	(0.08)	(0.09)	(0.20)
- No, not anymore	-0.01	-0.01	0.01	-0.01	-0.09	-0.02	-0.03	-0.02	0.01	-0.08
	(0.03)	(0.07)	(0.05)	(0.05)	(0.06)	(0.03)	(0.08)	(0.05)	(0.06)	(0.08)
Alcohol consumption: - Daily (Ref.: Never)	0.02	0.00	-0.01	0.05	0.02	0.03	0.04	0.01	0.04	0.05
	(0.05)	(0.22)	(0.12)	(0.08)	(0.10)	(0.07)	(0.28)	(0.16)	(0.10)	(0.13)
- Several times a week	0.00	0.05	-0.04	0.04	-0.06	0.00	-0.01	-0.02	0.03	-0.02
	(0.04)	(0.11)	(0.06)	(0.06)	(0.08)	(0.04)	(0.13)	(0.08)	(0.07)	(0.11)
- Once a week	-0.02	0.01	-0.06	0.01	-0.01	-0.01	0.01	-0.03	0.01	-0.04
	(0.04)	(0.09)	(0.06)	(0.07)	(0.11)	(0.04)	(0.11)	(0.08)	(0.08)	(0.12)
- 1-3 times a month	-0.01	0.01	-0.03	0.02	-0.11	-0.01	-0.03	-0.02	0.04	-0.15
	(0.04)	(0.08)	(0.06)	(0.07)	(0.10)	(0.04)	(0.10)	(0.07)	(0.08)	(0.11)
- Less often	0.01	0.07	-0.05	0.05	-0.07	-0.01	0.01	-0.06	0.03	-0.05
	(0.03)	(0.07)	(0.06)	(0.06)	(0.09)	(0.04)	(0.09)	(0.07)	(0.07)	(0.11)
Sports activities: Less than one hour a week (Ref.: No sports activity)	-0.05	-0.11	-0.03	-0.03	-0.10	-0.04	-0.09	-0.04	-0.04	0.00
	(0.03)	(0.09)	(0.06)	(0.06)	(0.08)	(0.04)	(0.10)	(0.07)	(0.07)	(0.11)

- Regularly, 1-2 hours a week	-0.08	-0.12	-0.07	-0.08	-0.14	-0.05	-0.07	-0.07	-0.05	-0.05
	(0.03)	(0.09)	(0.05)	(0.06)	(0.08)	(0.04)	(0.10)	(0.07)	(0.06)	(0.09)
- Regularly, 2-4 hours a week	-0.07	-0.11	-0.11	-0.00	-0.08	-0.04	-0.08	-0.10	0.05	-0.06
	(0.04)	(0.09)	(0.06)	(0.07)	(0.09)	(0.04)	(0.11)	(0.07)	(0.08)	(0.11)
- Regularly, more than 4 hours a week	-0.07	-0.07	-0.09	-0.06	-0.07	-0.08	-0.11	-0.12	-0.05	-0.08
	(0.04)	(0.10)	(0.07)	(0.07)	(0.11)	(0.04)	(0.12)	(0.08)	(0.07)	(0.12)
Chronic diseases: Presence of at least one chronic disease (Absence of chronic diseases)	-0.03	0.00	-0.08	0.03	-0.04	-0.00	0.00	-0.03	0.02	-0.00
	(0.02)	(0.06)	(0.04)	(0.04)	(0.07)	(0.03)	(0.08)	(0.05)	(0.05)	(0.08)
Self-rated health (1 = very bad to 5 = very good)	-0.26	-0.27	-0.26	-0.28	-0.16	-0.25	-0.23	-0.21	-0.31	-0.20
	(0.01)	(0.04)	(0.03)	(0.03)	(0.04)	(0.02)	(0.05)	(0.03)	(0.03)	(0.05)
Coronavirus anxiety (CAS)	0.14	0.13	0.15	0.10	0.17	0.21	0.16	0.28	0.16	0.10
	(0.00)	(0.01)	(0.01)	(0.01)	(0.01)	(0.00)	(0.01)	(0.01)	(0.01)	(0.02)
Pet ownership: No (Ref.: Yes)	-0.02	-0.04	-0.03	0.01	-0.03	0.01	-0.01	-0.01	0.05	-0.01
	(0.02)	(0.05)	(0.04)	(0.04)	(0.06)	(0.03)	(0.06)	(0.04)	(0.05)	(0.07)
Observations	3,091	577	1,076	995	443	3,091	577	1,076	995	443
R^2	0.21	0.24	0.24	0.23	0.26	0.25	0.24	0.29	0.25	0.23

Standardized beta-coefficients are reported; robust standard errors in parentheses
